# Simulation-based assessment of the P-glycoprotein expression-activity relationship shows a drug and system dependency

**DOI:** 10.1007/s10928-025-10015-6

**Published:** 2026-02-02

**Authors:** Daan W. van Valkengoed, Vivi Rottschäfer, Elizabeth C. M. de Lange

**Affiliations:** 1 Division of Systems Pharmacology and Pharmacy, Leiden Academic Centre for Drug Research, Leiden University, Leiden, The Netherlands; 2 Mathematical Institute, Leiden University, Leiden, The Netherlands; 3 Korteweg-de Vries Institute for Mathematics, University of Amsterdam, Amsterdam, The Netherlands

**Keywords:** P-glycoprotein, IVIVE, Expression-activity, BBB, Kinetic modelling

## Abstract

*In vitro-in vivo* extrapolation (IVIVE) of P-glycoprotein (P-gp) transporter activity bears two important assumptions: (1) P-gp expression (i.e., concentration) is linearly related to P-gp activity; and (2) this relation is drug independent. However, conflicting experimental results have been obtained about this relationship. This study therefore aimed to theoretically explore the P-gp expression-activity relationship (EAR). A P-gp membrane kinetic binding model was used to explore the P-gp EAR, and how this is impacted by different drugs as well as drug dose and initial P-gp expression. The model included passive permeability and drug interaction with P-gp described through the association (*k*_*on*_), dissociation (*k*_*off*_), and efflux rate (*k*_*e*_) constants. Simulations were first performed for 7 P-gp substrates, assuming an initial P-gp expression of 1000 µM and drug concentrations of 1 µM. Subsequently, the P-gp concentration was varied (2-300%) to derive the EAR. Next, to determine the impact of drug-specific and system-dependent properties on the P-gp EAR, virtual drugs varying in *k*_*off*_ and *k*_*e*_ were simulated, simultaneously considering different initial P-gp expressions and drug concentrations. Our simulations show that the P-gp EAR is not always linear, with the EAR showing both linear and non-linear behaviour depending on the drug. The *k*_*off*_*/k*_*e*_ ratio of a drug was found to be an important determinant of the P-gp EAR, which shifted towards non-linearity for lower *k*_*off*_/*k*_*e*_ ratios. Additionally, the P-gp EAR was more likely to be non-linear for higher ratios of the initial P-gp expression to drug concentration. In conclusion, this study shows that P-gp expression is not always proportional to P-gp activity, thereby explaining experimental contradictions about the EAR. This implies that using P-gp expression as single biomarker for P-gp activity needs to be reconsidered.

**Supplementary Information:**

The online version contains supplementary material available at 10.1007/s10928-025-10015-6.

## Introduction

Active transporters play a crucial role in determining a drug’s disposition in the body [1]. Through active influx or active efflux, they can increase or hamper a drug’s crossing of biological membranes, against the concentration gradient that drives passive diffusion. As such, transporters are crucial to ensure homeostasis and to protect organs from potentially harmful substances. The brain is an extremely well-protected organ due to the presence of the blood-brain-border (BBB) and the blood-cerebrospinal fluid-border (BCSFB) [2, 3]. Together with the paracellular tight junctions, active transporters at these borders control the exchange of compounds between the blood and the central nervous system (CNS). Specifically, the borders can impact both the rate (i.e., how quick) and extent (i.e., how much) of drug distribution to the CNS [4]. Consequently, CNS pharmacokinetic (PK) profiles may substantially deviate from the PK profiles observed in the systemic circulation. In addition, the PK can vary between different locations of the CNS itself [5]. Knowledge of the (unbound) PK profiles of CNS active drugs in the CNS, especially in the brain extracellular fluid (ECF), is however of crucial importance to develop and improve CNS disease treatment [6]. Direct sampling in the human brain is however highly restricted due to ethical considerations [5], and while it is possible to obtain limited CSF samples from the lumbar spinal region in humans, CSF concentrations may, especially for transporter substrates, not be representative of brainECF concentrations [7–9]. Overall, the complexity of the CNS and insufficient knowledge of the CNS PK of novel compounds is an important factor in the high attrition rates seen for CNS-based drugs [10–12].

Translational methods that make use of in vitro data to predict CNS drug disposition of transporter substrates in vivo (*in vitro-in vivo* extrapolation, IVIVE) are therefore of great importance for CNS drug development. The most commonly applied in vitro assay to derive quantitative information on efflux transporter activity is the transwell permeability assay, using a monolayer of cells expressing the transporter of interest. For CNS research, this commonly entails the study of the P-glycoprotein (P-gp) efflux transporter, but also breast cancer resistance protein (BCRP). Quantitative measures of both passive diffusion and active transport can be derived from this experimental set-up [13]. The degree of active efflux transport can be expressed as the efflux ratio (ER), which quantifies the asymmetric transport across the membrane caused by transporters. The ER has shown translational value to predict in vivo PK, for example in predicting the in vivo extent of drug distribution to the brain (K_p,uu,brain_) for P-gp and BCRP substrates [14–17].

K_p,uu,brain_ values, though informative about the extent of drug distribution to the brain, lump all BBB transport processes. Mechanistic insight into single transporter mediated drug transport (transporter-mediated clearance, CL) is important. To that end, CNS physiologically based PK (PBPK) models can be applied which give temporal predictions of drug PK [7, 18, 19]. Ideally, transporter-mediated PK in these models is also predicted using in vitro data, which has previously been explored to predict CNS PK profiles of P-gp substrates in different species [20–23].

A crucial question in IVIVE of transporter activity, is how to account for the differences in P-gp activity between systems, for example between a P-gp expressing cell line and P-gp at the human BBB. It is often assumed that P-gp protein expression is linearly related to its drug efflux activity [14, 24], in a drug-independent manner. This is the basis for scaling measures of activity, like the ER, between in vitro and in vivo systems with the use of a relative expression factor (REF) [13, 25, 26], defined as the ratio of P-gp protein expression in vivo to that in vitro.

Although scaling with the REF has been shown to be beneficial, there are conflicting results. In some in vivo, in vitro, and in silico investigations, a linear relationship between P-gp expression and its activity could not be found [22, 27–30]. In addition, multiple (CNS) PBPK approaches have been published which were unable to predict observed data by scaling P-gp activity in a bottom-up manner using expression differences, sometimes relying on empirical (drug-dependent) scaling factors [16, 22, 31–35]. This indicates that the general assumption of a linear relationship between P-gp expression and activity might not (always) hold. Being key information for robust IVIVE of P-gp activity, it is crucial to clarify to what extent P-gp expression relates to P-gp activity, and whether this relationship is drug-independent.

The aim of the current study is therefore to investigate the P-gp expression-activity relationship (P-gp EAR) from a more theoretical viewpoint. To that end we performed simulations using a P-gp kinetic membrane model originally derived by Tran et al. [36], which is based on transwell permeability assay data [36–38]. The simulations were done for 7 well-known P-gp substrates (amprenavir, digoxin, ketoconazole, loperamide, quinidine, verapamil, and vinblastine) and subsequently expanded to virtual drugs to obtain a more fundamental understanding of the P-gp EAR. Specifically, we investigated how both differences in drug-specific properties and changes in initial conditions like P-gp expression and drug concentration influence the P-gp EAR. Finally, we relate these findings to experimental observations on transporter EAR.

## Methods

### Model overview

**Fig. 1:**
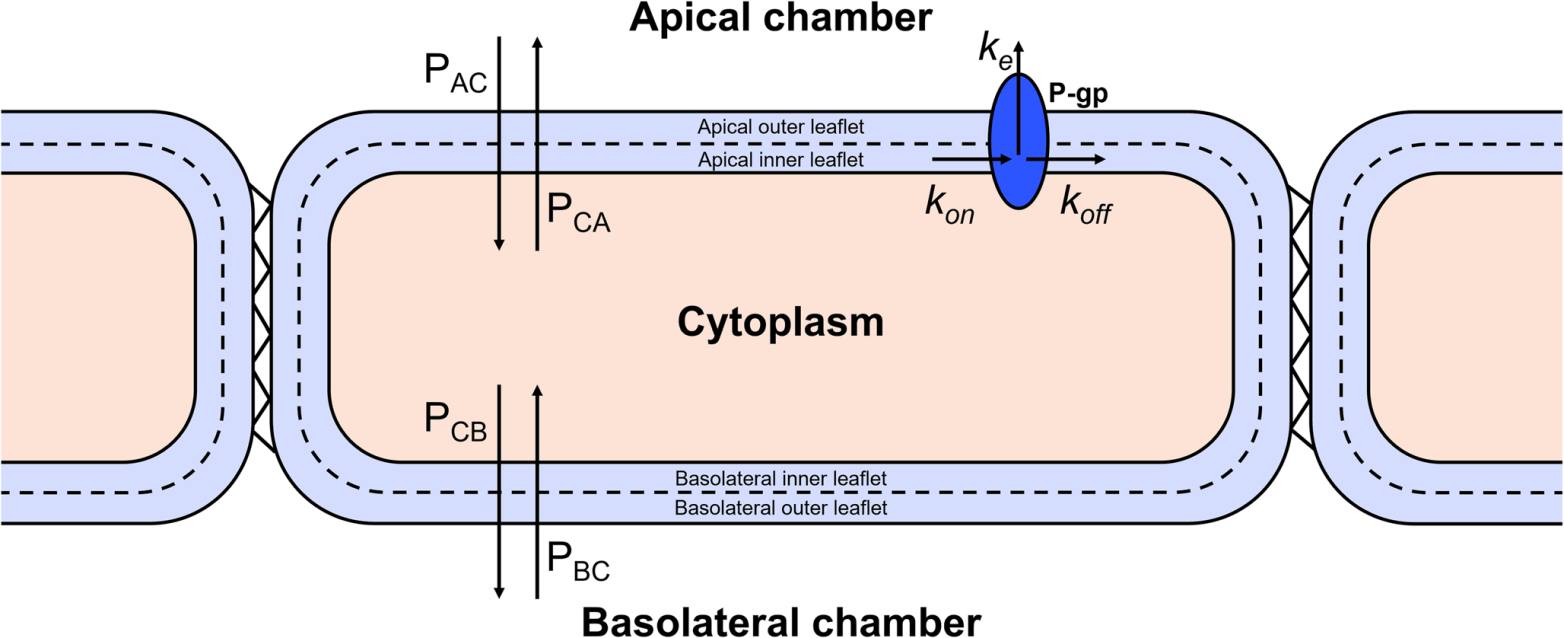
**Schematic representation of the P-gp kinetic model** [36]. The model represents the in vitro setup commonly used for transwell permeability assays. Here, a monolayer of a P-gp expressing cell line is used to determine the permeability of compounds across the monolayer. The drug is dosed at either the apical or the basolateral chamber, after which the drug concentration in the receiver chamber is measured over time. From this, information on the drug’s rate of diffusion as well as its interaction with P-gp can be derived. In the model, a drug can passively diffuse across the membranes to and from the cytoplasm. P_AC_= the passive permeability coefficient for drug diffusion from the apical side to the cytoplasm. P_CA_= the passive permeability coefficient for drug diffusion from the cytoplasm to the apical side. P_BC_= the passive permeability coefficient for drug diffusion from the basolateral side to the cytoplasm. P_CB_= the passive permeability coefficient for drug diffusion from the cytoplasm to the basolateral side. Whenever drug is present in the inner leaflet of the apical membrane, it can bind to P-gp with the association rate constant (k_on_). Subsequently, it can either be effluxed to the apical chamber (with the efflux rate constant k_e_) or dissociate back into the inner leaflet with the dissociation rate constant (k_off_)

Simulations of P-gp activity were performed using a mass-action kinetic model for P-gp efflux that was derived in Tran et al. [36], as schematically represented in Fig. 1. It resembles the common experimental transwell permeability assay for measuring in vitro drug permeability across a monolayer of cells expressing P-gp. Here, a drug is dosed at either the apical chamber (AC) or the basolateral chamber (BC), after which the drug concentration on the other side of the membrane is measured over time. From this, quantitative measures on the passive diffusion rate (*P*) and P-gp mediated transport can then be determined [39]. As this situation closely resembles the in vivo situation at the BBB, where a monolayer of brain endothelial cells separates blood (apical chamber) from the brainECF (basolateral chamber), the transwell permeability assay is a common choice for IVIVE based prediction of P-gp mediated transport at the BBB [14–17].

The model derived in Tran et al. describes the variation of drug concentrations in the apical chamber, the basolateral chamber, the cytoplasm and the inner and outer leaflets of both membranes separately [36]. After a drug is dosed in either the apical or basolateral chamber, the drug can diffuse through the monolayer and can interact with P-gp, after which it could be effluxed to the apical chamber.

Drugs diffuse through the membrane according to their passive diffusion rate *P* (expressed in dm/s), which can be multiplied by the surface area (SA) of the membrane to obtain a passive diffusion clearance. P_AC_ and P_CA_ represent the diffusion rate from the apical chamber into the cytoplasm and vice versa, and P_BC_ and P_CB_ the passive diffusion rate from the basolateral chamber into the cytoplasm and vice versa. P_AC_ and P_BC_ may have different values, however, here we assume that the diffusion rates across the apical and basolateral membranes are equal, so that:

1 $$\:\begin{array}{c}P_{AC}=P_{CA}\end{array}$$

2 $$\:\begin{array}{c}P_{BC}=P_{CB.}\end{array}$$

Although P-gp is a transmembrane protein, its binding site is specifically located in the inner leaflet of the apical membrane [40]. As such, only the drug present in the apical inner leaflet is assumed to interact with P-gp. Drug binding to P-gp is described through the association rate constant *k*_*on*_ (expressed in 1/M/s). Then, after binding to P-gp, drugs can either dissociate back into the inner leaflet with a dissociation rate constant *k*_*off*_ (expressed in 1/s) or they can be effluxed to the apical chamber by P-gp, described with the efflux rate constant *k*_*e*_ (expressed in 1/s).

The major assumption under which the model in Tran et al. is derived, is that instantaneous equilibration occurs between each lipid monolayer and the aqueous compartment directly facing it. As such, the apical chamber is in instantaneous equilibrium with the apical outer leaflet, the cytoplasm with both the apical and basolateral inner leaflets, and the basolateral chamber with the basolateral outer leaflet [36]. The extent of partitioning between the lipid monolayer and the aqueous compartment is described through a drug’s partition coefficient (*K*). The kinetic model uses three partition coefficients: K_AO_, K_IL_ and K_BO_, representing partitioning into the apical outer leaflet, the inner leaflets and the basolateral outer leaflet, respectively. They are defined as:

3 $$\:\begin{array}{c}K_{AO}=\frac{C_{AO}}{C_{AC}}\end{array}$$

4 $$\:\begin{array}{c}K_{IL}=\frac{C_{IL}}{C_{CP}}\end{array}$$

5 $$\:\begin{array}{c}K_{BO}=\frac{C_{BO}}{C_{BC}},\end{array}$$

where C_AO_, C_IL_ and C_BO_ are the lipid phase drug concentrations in the apical outer leaflet, inner leaflets and basolateral outer leaflet, and C_AC_, C_CP_ and C_BC_ are the drug concentrations in the apical chamber, cytoplasm and basolateral chamber, respectively. Following the assumption of instantaneous equilibrium, the drug concentration in each lipid phase (e.g., in the inner leaflet, C_IL_) is then equal to the concentration in the aqueous phase facing the lipid phase multiplied by the relevant partition coefficient (e.g., C_IL_ = C_CP_K_IL_).

The kinetic model also accounts for unexplained loss from the system that might be observed for some drugs in vitro. It is assumed that the drug is removed with a rate constant *k*_*v*_ (expressed in 1/s). In our simulations, we assume very little loss, and fixed *k*_*v*_ to 1*10^− 6^/s, as described for amprenavir (which had negligible loss) in Tran et al. Following these modelling assumptions, the kinetic model is given by a system of ordinary differential equations (ODEs) [36]:

6 $$\:\begin{array}{c}V_{A,tot}\frac{{dC}_{AC}}{dt}=P_{AC}{SA}_A\left(C_{CP}-C_{AC}\right)-\:k_vV_{A,tot}C_{AC}+{0.5V}_{IL}k_eC_{Pgp,bound}\end{array}$$

7 $$\begin{aligned}V_{C,tot}\frac{{dC}_{CP}}{dt}&=P_{AC}{SA}_A\left(C_{AC}-C_{CP}\right)\\&+\:P_{BC}{SA}_B\left(C_{BC}-C_{CP}\right)\:-\:k_vV_{C,tot}C_{CP}\end{aligned}$$

$$\:-{0.5V}_{IL}{k}_{on}{K}_{IL}{C}_{CP}{C}_{Pgp,free}+{0.5V}_{IL}{k}_{off}{C}_{Pgp,bound}$$

8 $$\:\begin{array}{c}V_{B,tot}\frac{{dC}_{BC}}{dt}=P_{BC}{SA}_B\left(C_{CP}-C_{BC}\right)-\:k_vV_{B,tot}C_{BC}\end{array}$$

9 $$\:\begin{array}{c}\frac{{dC}_{Pgp,free}}{dt}=\:-k_{on}K_{IL}C_{CP}C_{Pgp,free}+\left(k_{off}+k_e\right)C_{Pgp,bound}\end{array}$$

10 $$\:\begin{array}{c}\frac{{dC}_{Pgp,bound}}{dt}=\:k_{on}K_{IL}C_{CP}C_{Pgp,free}-\left(k_{off}+k_e\right)C_{Pgp,bound}\end{array}$$

where C_AC_, C_CP_, and C_BC_ are the drug concentrations in the apical chamber, cytoplasm and basolateral chamber, respectively. C_Pgp,free_ and C_Pgp,bound_ correspond to the free and bound P-gp concentrations. Because of the assumption of instantaneous equilibration, the volumes used in the ODEs are defined as V_A,tot_, V_B,tot_ , and V_C,tot_ which represent, respectively, the whole apical, basolateral or cytoplasmic volume accessible to the drug, as given by:

11 $$\:\begin{array}{c}V_{A,tot}=V_A+K_{AO}V_{AO}\end{array}$$

where V_A_ is the apical chamber volume, V_AO_ is the volume of the apical outer leaflet and K_AO_ is the partition coefficient between the apical chamber and the apical outer leaflet. Similar expressions are found for V_B,tot_ and V_C,tot_:

12 $$\:\begin{array}{c}V_{B,tot}=V_B+K_{BO}V_{BO}\end{array}$$

13 $$\:\begin{array}{c}V_{C,tot}=V_C+K_{IL}V_{IL}\end{array}$$

We assume that at the start of the simulation (*t* = 0), there is no drug in the system, apart from in the apical chamber. Therefore

14 $$\:\begin{array}{c}C_{AC}\left(0\right)=Dose\end{array}$$

15 $$\:\begin{array}{c}C_{CP}\left(0\right)=0\end{array}$$

16 $$\begin{array}{c}C_{BC}\left(0\right)=0\end{array}$$

17 $$\:\begin{array}{c}C_{Pgp,bound}\left(0\right)=\:0\end{array}$$

18 $$\:\begin{array}{c}\:C_{Pgp,free}\left(0\right)=\:C_{Pgp,tot}\end{array}$$

where *Dose* is the administered drug dose, and $\:{C}_{Pgp,tot}\:$represents the total P-gp concentration in the system. Note that at any given moment, *C*_*Pgp*_,_*free*_ + *C*_*Pgp*_,_*bound*_ = $\:{C}_{Pgp,tot}$.

An overview of all the parameters, variables, explanations and units used in the model are given in Table 1.

**Table 1:** Overview of all variable and parameter abbreviations as used in the kinetic binding model, their descriptions, the units used in the model and their value if it is a fixed value

Abbreviation	Description	Unit in model	Fixed value (from [36])
Variables			
C_AC_	Concentration in apical chamber	mole/L (M)	-
C_BC_	Concentration in basolateral chamber	mole/L (M)	-
C_CP_	Concentration in cytoplasm	mole/L (M)	-
C_Pgp,free_	Free P-gp concentration	mole/L (M)	-
C_Pgp,bound_	P-gp concentration bound to drug	mole/L (M)	-
C_Pgp,tot_	Total P-gp concentration in the system, equal to C_Pgp, free_ + C_Pgp, bound_	mole/L (M)	-
t_end_	End time of the simulations used to determine P-gp EAR	Seconds (s)	21,600
**Drug-specific parameters**			
K_AO_	Partition coefficient of drug from apical chamber into apical membrane outer leaflet	Unitless	-
K_BO_	Partition coefficient of drug from basolateral chamber into basolateral membrane outer leaflet	Unitless	-
K_IL_	Partition coefficient of drug from cytoplasm into plasma membrane inner leaflet	Unitless	-
*k*_*e*_	Efflux rate constant	1/s	-
*k*_*off*_	Dissociation rate constant	1/s	-
*k*_*on*_	Association rate constant	1/M/s	-
*k*_*v*_	Rate constant for unexplained loss from the system	1/s	1*10^− 6^
P_AC_	Passive permeability coefficient from apical chamber to cytoplasm. Assumed to be equal to P_CA_	dm/s	-
P_BC_	Passive permeability coefficient from basolateral chamber to cytoplasm. Assumed to be equal to P_CB_	dm/s	-
**System-specific parameters**			
SA_A_	Surface area of apical membrane	dm^2^	2.26*10^− 2^
SA_B_	Surface area of basolateral membrane	dm^2^	2.26*10^− 2^
V_A_	Volume of apical chamber	dm^3^	5*10^− 4^
V_AO_	Volume of apical membrane outer leaflet	dm^3^	5*10^− 10^
V_Atot_	Apical volume accessible to drug	dm^3^	-
V_B_	Volume of basolateral chamber	dm^3^	1.5*10^− 3^
V_BO_	Volume of basolateral membrane outer leaflet	dm^3^	5*10^− 10^
V_B,tot_	Basolateral volume accessible to drug	dm^3^	-
V_C_	Volume of the cytoplasm	dm^3^	1*10^− 6^
V_IL_	Total volume of the two inner leaflets facing cytoplasm	dm^3^	1*10^− 9^
V_Ctot_	Cytoplasmic volume accessible to drug	dm^3^	-

### Determining the P-gp expression-activity relationship (EAR)

As a measure of P-gp activity we use the amount of a drug that is effluxed by P-gp during the simulation. At any given timepoint *t*, the amount (moles) of a drug effluxed by P-gp (A_effluxed_) is given as:

19 $$\:\begin{array}{c}A_{effluxed}\left(t\right)=\:k_eC_{Pgp,bound}\left(t\right){0.5V}_{IL}\end{array}$$

where *k*_*e*_ is the efflux rate constant, C_Pgp, bound_*(t)* is the concentration of P-gp bound to drug in the apical inner leaflet at timepoint *t* (see Eq. 10), and 0.5*V_IL_ is the volume of the apical inner leaflet, since V_IL_ represents the total volume of both inner leaflets.

We are interested in the cumulative amount of drug effluxed from the start (*t = 0*) until time t_end_, which we denote as $\:{A}_{eff,cum}\left({t}_{end}\right)$. This can be determined as

20 $$\:\begin{array}{c}A_{eff,cum}\left(t_{end}\right)={\int\:}_0^{t_{end}}A_{effluxed}\left(t\right)dt\end{array}$$

In our simulations $\:{t}_{end}$ was set to 6 h (21600 s), to correspond to the experiments by Tran et al.

To determine the P-gp EAR, two simulations are done. First, one at a reference P-gp expression (e.g., 1000 µM, which is set as 100%), and then one with a different P-gp expression (*x*%). The *relative* P-gp activity at x% P-gp expression (rEAR_x%_) is then described as:

21 $$\:\begin{array}{c}{\mathrm{r}\mathrm{E}\mathrm{A}\mathrm{R}}_{\mathrm{x}\mathrm{\%}}=\:\frac{A_{eff,cum,x\%\:Pgp\:\left(6\right)}}{A_{eff,cum,100\%\:Pgp\:\left(6\right)}}\ast100\%\end{array}$$

**Fig. 2:**
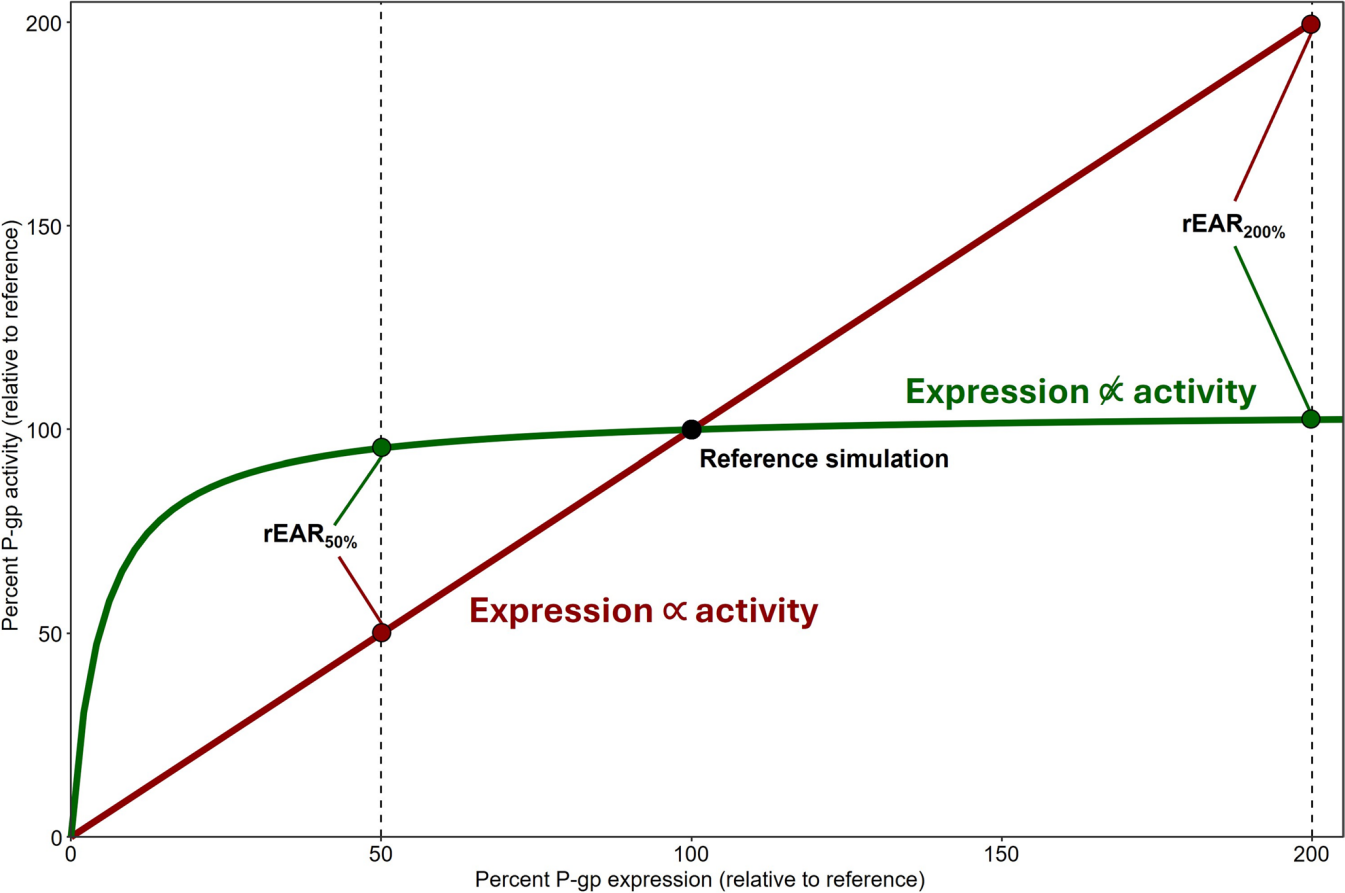
**Visual guide on the rEAR**_**x%**_. The black dot indicates the reference simulation with 100% P-gp expression. The red line represents a linear EAR, and the red points indicate such a linear EAR, where rEAR_50%_ = 50%, and rEAR_200%_ = 200%. The green line showcases a strongly non-linear EAR where rEAR_50%_ = 95%, and rEAR_200%_ = 102%

If rEAR_x%_ = *x%*, this means that the P-gp EAR is considered linear. Whenever rEAR_x%_ deviates from *x%*, the P-gp EAR moves towards non-linearity (see Fig. 2 for a visual guide on interpreting the rEAR_x%_).

### Determining the EAR for P-gp substrates

Simulations of the P-gp EAR were first performed for a set of seven P-gp substrates (amprenavir, digoxin, ketoconazole, loperamide, quinidine, verapamil and vinblastine). The kinetic parameters of these compounds were estimated previously in Lumen et al. [38], and are provided in Table 2. The partition coefficients between the lipid and aqueous phases (i.e., K_AO_, K_BO_, K_IL_) were previously experimentally determined in unilamellar liposomes with membrane lipid compositions that match the general composition of the apical and basolateral inner and outer leaflets [38].

**Table 2:** Parameter values reported by Lumen et al. [38] for seven P-gp substrates. All parameters were fitted to experimental in vitro data, except for the partitioning coefficients (K_AO_, K_BO_ and K_IL_) that were derived from in vitro experiments

Drug	k_on_ (1/M/s)	k_off_ (1/s)	k_e_ (1/s)	*P*_AC_ (nm/s)	*P*_BC_ (nm/s)	K_AO_	K_BO_	K_IL_
Amprenavir	1 * 10^8^	7 * 10^4^	30	350	420	150	200	100
Digoxin	1 * 10^8^	3 * 10^4^	3	40	50	100	100	100
Ketoconazole	1 * 10^8^	3 * 10^4^	0.2	730	680	400	400	1000
Loperamide	1 * 10^8^	2 * 10^4^	0.4	320	320	300	450	1500
Quinidine	1 * 10^8^	4 * 10^3^	3	670	670	100	100	350
Verapamil	1 * 10^8^	2 * 10^4^	0.1	540	580	200	300	650
Vinblastine	1 * 10^8^	5 * 10^4^	3	55	90	200	200	200

To obtain the P-gp EAR for these drugs, simulations were performed assuming administration of 1 µM drug at *t* = 0 in the apical chamber. The reference P-gp expression in these simulations (i.e., 100% P-gp) was equal to the P-gp expression estimated by Lumen et al. in MDCKII-MDR1 cells, namely 1000 µM. The rEAR_x%_ was subsequently determined at P-gp expression levels of 2%, 7%, 15%, 30%, 50%, 200% and 300% of the reference P-gp concentration for each drug, from which the EAR for these drugs under the given dose and initial P-gp expression was determined.

### Determining the relative P-gp activity at 50% P-gp expression (rEAR_50%_) for virtual drugs

To better understand the P-gp EAR, we simulated the model across a wide range of model parameter values and their respective combinations. For these simulations, P_AC_ and P_BC_ were assumed to be equal. The kinetic parameters *k*_*off*_ and *k*_*e*_ were varied ranging from 10^2^ to 10^7^ s^− 1^ (*k*_*off*_) and 0.03–30 s^− 1^ (*k*_*e*_). Moreover, the drug concentrations in the apical chamber (0.1–100 µM) and reference P-gp expression (10^1^ – 10^5^ µM) were also varied. Every unique combination of the values was then simulated, and thereafter rEAR_50%_ was obtained. The rEAR_50%_ was used as a measure to distinguish a linear from a non-linear P-gp EAR, but in principle other values could also be chosen to analyse this.

### Software

The P-gp kinetic binding model was simulated in RStudio [41] running R version 4.3.3, using the freely available R package for solving of ODE systems, rxode2 version 3.0.4 using the LSODA ODE solver. For data visualization, the R package ggplot2 version 3.5.2 was used.

## Results

### The P-gp EAR is drug dependent

**Fig. 3:**
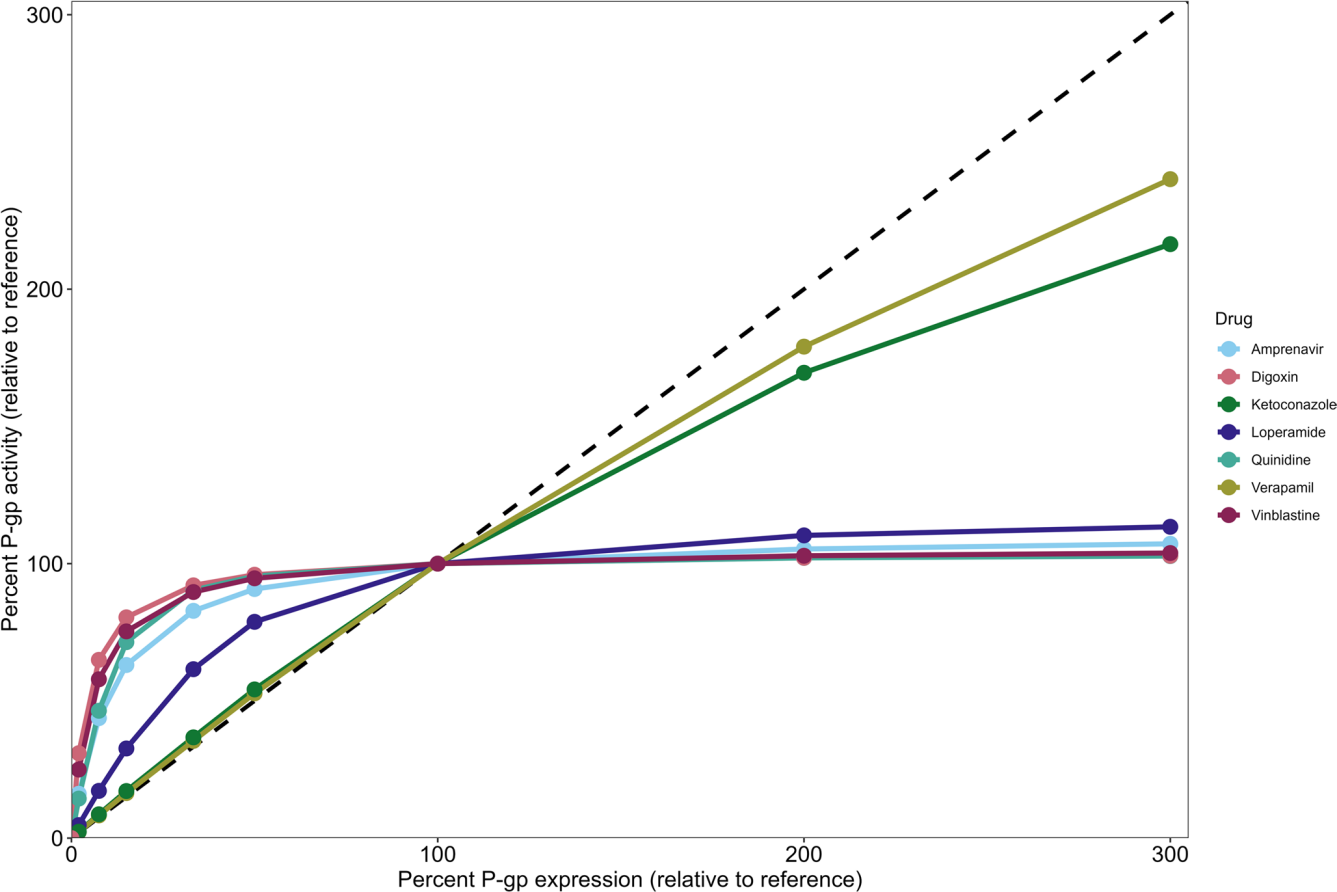
P-gp expression-activity relationship (EAR) of seven P-gp substrates. All the simulations started with a reference (100%) P-gp expression of 1000 µM and the drug concentration in the apical chamber at t = 0 was 1 µM. P-gp expression was subsequently varied, and the relative change in activity (rEAR) was calculated as described in Sect. 2.2. The black dashed line represents a linear P-gp EAR

The P-gp EAR was first investigated for the 7 P-gp substrates for which estimated kinetic parameters were reported in Lumen et al. [38], as given in Table 2. In Fig. 3 we give the P-gp EAR for these substrates at 1 µM concentration and reference P-gp expression of 1000 µM. It shows that, as P-gp expression decreases, 4 drugs (amprenavir, digoxin, quinidine and vinblastine) show a distinctly non-linear P-gp EAR, with a rEAR_50%_ ranging between 91% and 96%. 2 drugs (ketoconazole and verapamil) display an almost linear P-gp EAR with decreasing P-gp expression, with rEAR_50%_ of 54% (ketoconazole) and 53% (verapamil). Loperamide shows an intermediate position between the non-linearly and linearly responding groups, with rEAR_50%_ of 79%.

When P-gp expression is increased, efflux activity of the non-linear compounds does not increase greatly compared to the reference condition. Quinidine, for example, shows a rEAR_300%_ of 103%. The P-gp EAR for loperamide is non-linear when increasing P-gp expression. For verapamil and ketoconazole, the P-gp EAR shows an increase in activity with increasing expression, however it becomes more non-linear with increasing P-gp expression. At 300% expression, verapamil shows a rEAR_300%_ of 240% and ketoconazole a rEAR_300%_ of 216%. Overall, with the exact same system-specific conditions (dose and initial P-gp expression), different drugs show distinct P-gp EARs.

**Fig. 4:**
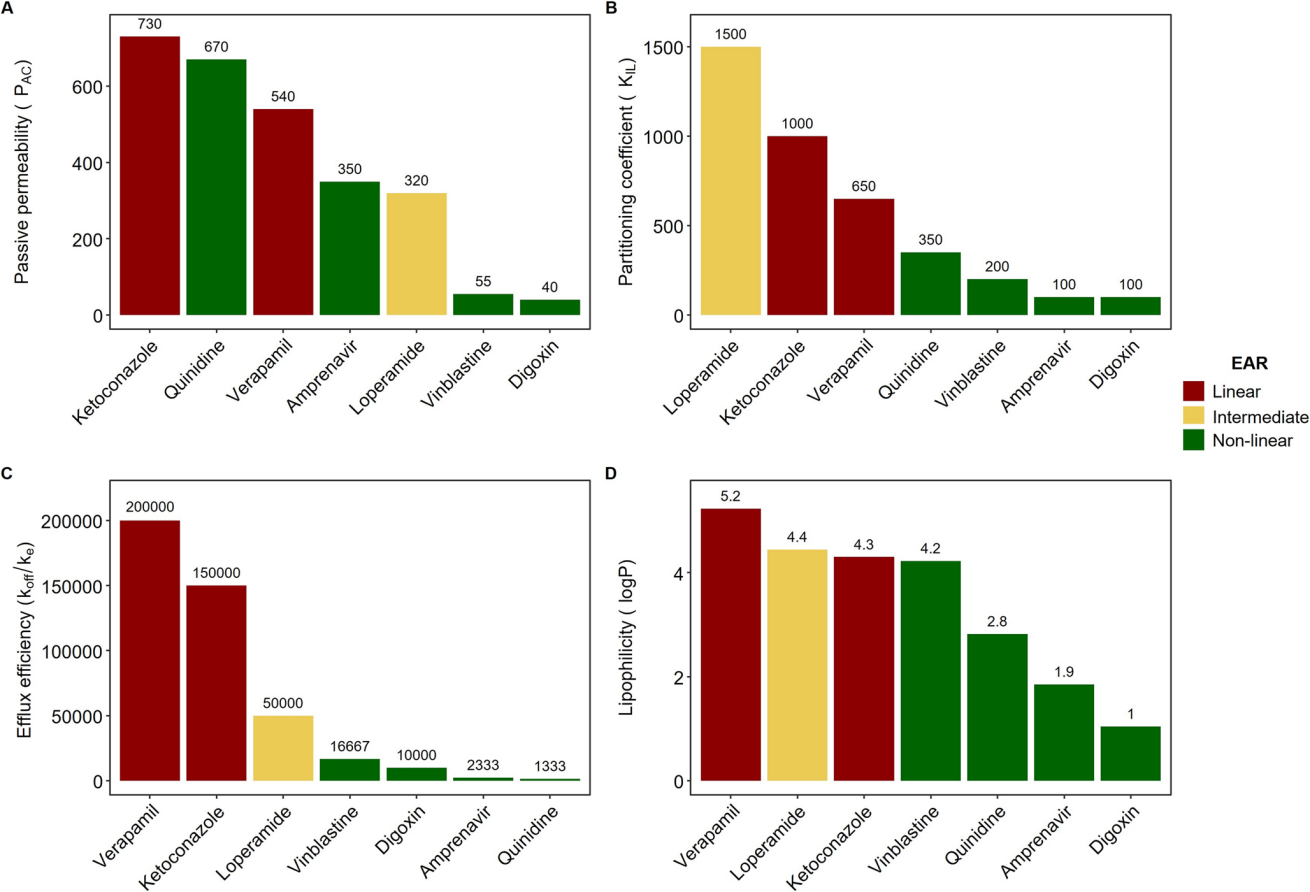
P-gp EAR for seven P-gp substrates and associated individual drug properties.. (**A**) PAC, (**B**) KIL, (**C**) efflux efficiency, (ratio koff/ke), and, (**D**) logP. Red bars indicate drugs that show a linear P-gp EAR, (verapamil and ketoconazole), green bars drugs with a non-linear P-gp EAR, (amprenavir, digoxin, quinidine and vinblastine), and yellow bars a drug with an intermediate P-gp EAR, (loperamide). The P-gp EARs were determined through simulations with P-gp expression of 1000 µM and apical drug concentration at t = 0 of 1 µM, (see Fig. 3)

To explore drug related properties responsible for the drug-dependent P-gp EAR, the relationship between each drug’s passive permeability across the apical membrane (P_AC_), partitioning into the inner leaflet (K_IL_), P-gp efflux efficiency (ratio *k*_*off*_/*k*_*e*_) and lipophilicity (logP) was related to the type of P-gp EAR (linear, intermediate, non-linear), as shown in Fig. 4. It shows that the only correlation between P-gp EAR and a drug property can be seen for the drug efflux efficiency, the ratio *k*_*off*_/*k*_*e*_. Similar to the individual values, the ratio of P_AC_ and K_IL_ also does not show a clear correlation to the type of EAR (supplementary Figure S1).

### Passive permeability and the partitioning coefficient impact but do not determine the P-gp EAR

**Fig. 5:**
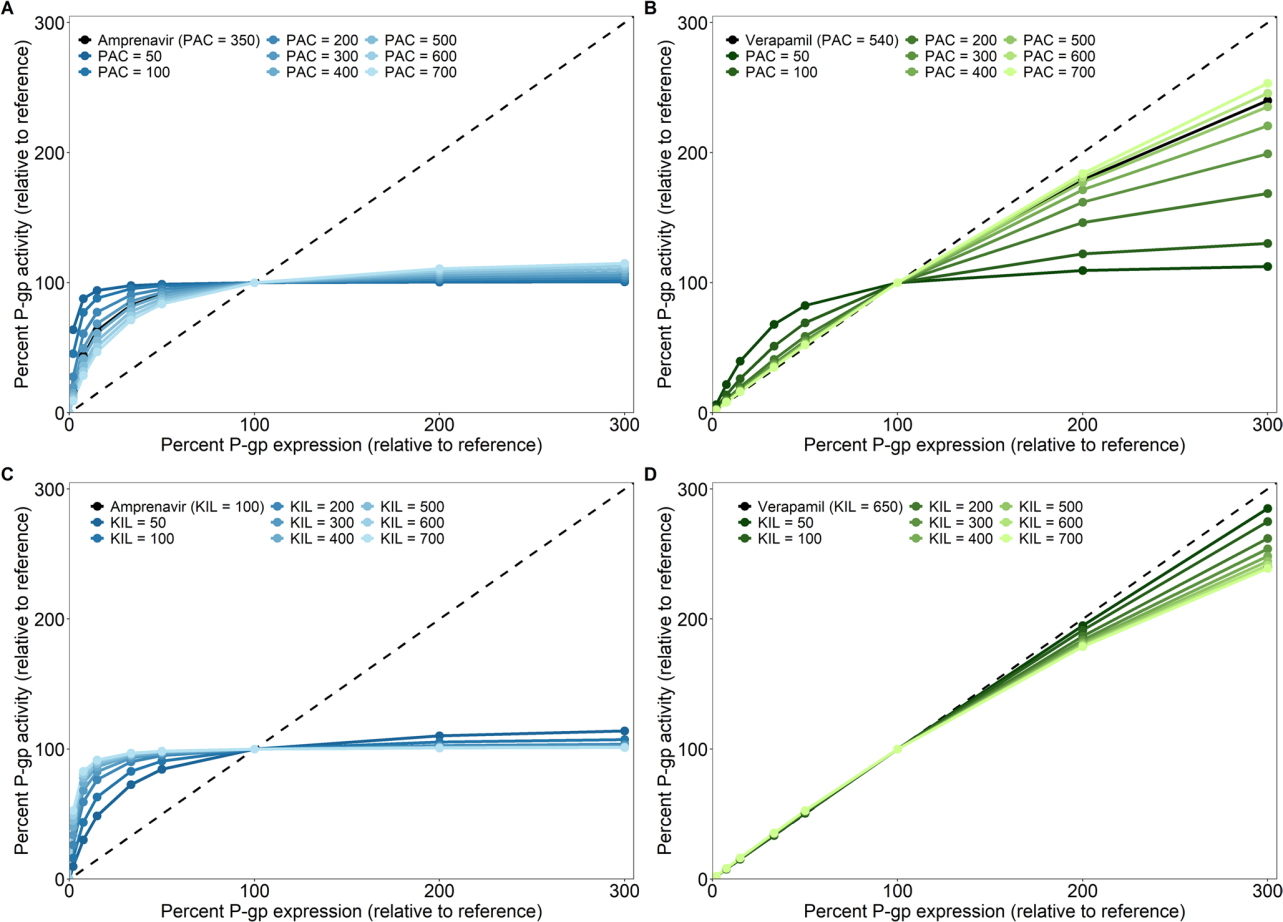
P-gp EAR for amprenavir-like (**A** and **C**) and verapamil-like (**B** and **D**) compounds where either the passive permeability P_AC_ (**A** and **B**) or the partitioning coefficient K_IL_ (**C** and **D**) is varied. In these simulations, the passive permeabilities at the apical and basolateral membranes are set to be equal so that P_AC_ = P_BC_, and are given in nm/s. The other parameters correspond to those estimated for amprenavir or verapamil by Lumen et al. (Table 2). Lighter colours indicate higher values of passive permeability or K_IL_. All the simulations started with a reference (100%) P-gp expression of 1000 µM and the apical drug concentration at t = 0 was 1 µM. The black dashed line represents a fully linear P-gp EAR

Even though the P_AC_ and K_IL_ do not seem to correlate to the observed P-gp EARs, we still wanted to determine to what extent they impact P-gp EARs. Figure 5 shows simulations for compounds which have the kinetic parameters of amprenavir (a drug with non-linear P-gp EAR, 5 A and 5 C) and verapamil (a drug with linear P-gp EAR, 5B and 5D), but with different passive permeability P_AC_ (Fig. 5A and B, in these simulations we assumed P_AC_ = P_BC_) or K_IL_ (Fig. 5C and D). From Fig. 5 we conclude that varying the passive permeability or K_IL_ does influence the P-gp EAR, however, the drugs do not switch completely from non-linear to linear P-gp EAR or vice versa. Amprenavir always shows a non-linear P-gp EAR, regardless of the passive permeability and K_IL_ (Fig. 5A and C). For verapamil only a strong decrease in passive permeability, around 5 to 10-fold, modifies the P-gp EAR to become more non-linear (Fig. 5B). Changes in K_IL_ for verapamil are of little influence (Fig. 5D).

To support the conclusion that the passive permeability and K_IL_ themselves were not the reason for difference in the P-gp EAR between amprenavir and verapamil, we also simulated a simultaneous change of passive permeability and partitioning coefficients for virtual drugs. These were created by changing both the PAC and partitioning coefficients of amprenavir to those of verapamil (creating “ampramil”), and vice versa for verapamil (creating “veravir”) while keeping the kinetic parameters of each drug the same (supplementary Figure S2). EAR simulations of these drugs show a P-gp EAR of ampramil that is more non-linear than that of amprenavir, whereas veravir shows a more linear P-gp EAR than that of verapamil. As such, although passive permeability and K_IL_ influence the P-gp EAR, they were not the reason for the drug-specific EARs observed in Fig. 3. 

### The ratio *k*_*off*_/*k*_*e*_, as well as the reference P-gp expression and drug concentration, determines the P-gp EAR

**Fig. 6:**
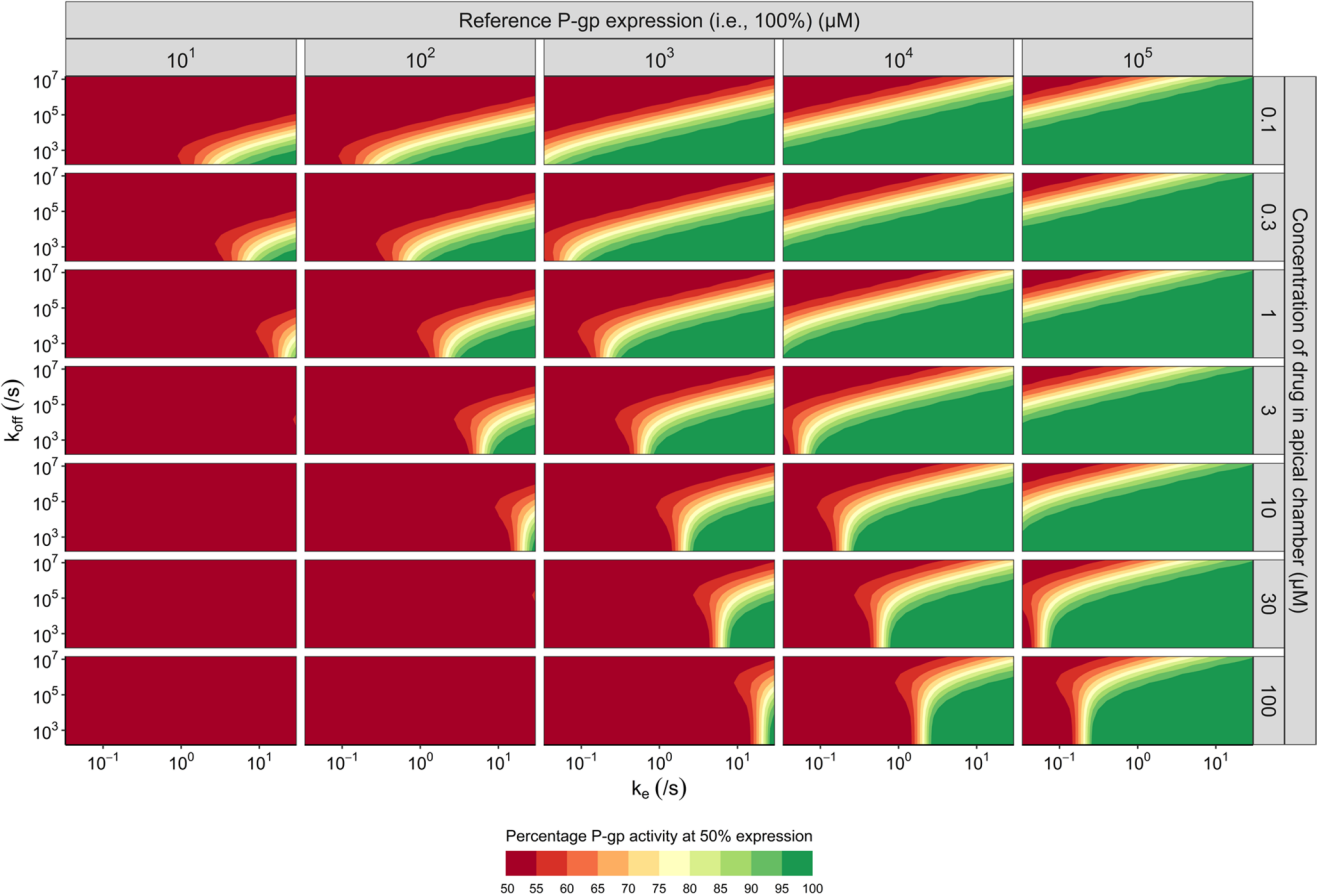
Heatmap of the rEAR_50%_ for virtual drugs with different combinations of *k*_*off*_ and *k*_*e*_ (axes), and different initial drug and reference P-gp expressions (rows and columns). In every figure, the value of *k*_*e*_ is plotted on the horizontal axis and the value of *k*_*off*_ is plotted on the vertical axis. The columns indicate the reference P-gp expression (i.e., 100%) (µM), whereas the rows indicate the drug concentration in the apical chamber at t = 0 (µM). Red colours indicate a linear EAR for 50% reduction in P-gp expression (rEAR_50%_ = 50%), while green corresponds to non-linear EAR (rEAR_50%_ = 100%). In these simulations, P_AC_ = P_BC_ = 300 nm/s, and K_AO_ = K_BO_ = K_IL_ = 300

To explore how the type of the P-gp EAR (i.e., non-linear or linear) changes with the kinetic parameters *k*_*e*_ and *k*_*off*_, a large set of virtual drugs was simulated. For all of these drugs we chose a passive permeability P_AC_ = P_BC_ = 300 nm/s and partitioning coefficients into membranes of K_AO_ = K_BO_ = K_IL_ = 300. Aside from the drug-specific properties, we simulated different reference P-gp expressions (1*10^1^−1*10^5^ µM) and varied the starting concentration of the drug (0.1–100 µM). For each unique condition, rEAR_50%_ was determined and plotted in Fig. 6. The heatmaps indicate a linear rEAR_50%_ with a red colour, whereas a non-linear rEAR_50%_ is shown in green.

As observed in Fig. 6, the relationship between *k*_*off*_ and *k*_*e*_ is an important driving factor in determining the P-gp EAR. In general, increasing *k*_*e*_ moves the P-gp EAR towards non-linearity (rEAR_50%_ = 100%), whereas increasing *k*_*off*_ moves the P-gp EAR towards linearity (rEAR_50%_ = 50%). Additionally, a P-gp EAR is not only determined by drug-specific characteristics, but also by the initial conditions (i.e., reference P-gp expression and drug concentration). Non-linear P-gp EAR have a higher chance to occur when the reference P-gp expression is higher than the initial drug concentration.

Simulations with a higher passive permeability of 700 nm/s show similar trends as observed in Fig. 6, however the EAR shifts to the right (i.e., a linear rEAR_50%_ is observed for a larger range of *k*_*off*_ and *k*_*e*_) (supplementary Figure S3). Increasing the K_IL_ shifts the EAR slightly upwards (i.e., towards more non-linear P-gp EAR) (supplementary Figure S4).

## Discussion

Successful translation of in vitro data to predict drug PK in vivo is challenging. Advances in IVIVE, especially by using mass spectrometry-based targeted proteomics to determine transporter expression, have given rise to mechanistic PBPK models with promising predictive and translational capabilities [13]. IVIVE of transporter activity assumes that transporter expression is linearly related to its activity, while also being drug and drug concentration independent. The use of expression-based scaling factors (REF) have improved the IVIVE approach, for example for translation of efflux ratios (ER) determined in vitro to in vivo Kp_uu_ values [14]. The REF has also been applied within PBPK models for temporal PK predictions [42–44]. However, the REF does not always lead to accurate IVIVE [13, 22, 31, 45–48]. In this study, we explored the P-gp expression-activity relationship (P-gp EAR) of several P-gp substrates and virtual drugs, using a P-gp kinetic binding model, and found drug, initial P-gp expression (i.e., concentration) and drug concentration dependencies. Our simulations indicate the need to reconsider the widespread use of P-gp expression as single biomarker for its activity.

The use of the REF to translate transporter activity is supported by some in vitro studies that have correlated expression of transporters to their functional activity. Kumar et al. have shown a proportionality between transporter expression and activity for the uptake transporter OATP1B1 and the efflux transporter BCRP [24]. In this study, transporter expression was normalized to Na^+^-K^+^ ATPase expression to ensure that the measure of expression reflects plasma membrane transporter expression only. However, they only investigated the transporter EAR for one substrate per transporter (uptake of estradiol-glucuronide for OATP1B1 and efflux of Hoechst 3342 for BCRP). As such, the transporter EAR cannot be generalized to other compounds. Liu et al. reported the relationship between the corrected efflux ratio (ER_c_) and BCRP expression in MDCKII cells for 4 BCRP substrates [49], and reported a linear relationship between BCRP expression and ER_c_. However, this relationship was described with distinct equations for each drug, differing in both the intercepts and slopes. We plotted their data together in one figure so that the drugs can be compared (supplementary Figure S5) and found that the BCRP EAR depends on the drug.

Storelli et al. reported the relationship between P-gp expression and in vitro ER for the P-gp substrates verapamil, N-desmethyl loperamide and metoclopramide in vitro [16], and concluded the EAR to be linear. However, as the relative changes in P-gp expression were too small for 2 of the 3 drugs (verapamil and metoclopramide), a proper relationship could not be demonstrated. Only N-desmethyl loperamide showed a non-linear increase in ER as P-gp expression increased. Tachibana et al. investigated the relationship between J_max_, the maximum P-gp activity, and P-gp expression using Caco-2 and MDKCII-MDR1 cells for multiple compounds, namely quinidine, verapamil and vinblastine [50]. There, a linear relationship between P-gp expression and J_max_ was reported. However, similar as in Liu et al. [49], these relationships were drug dependent. When only considering the J_max_ derived from Caco-2 cells and normalizing the data to allow better comparison between the drugs (supplementary Figure S6), we observed that only verapamil’s J_max_ is linearly related to P-gp expression. Quinidine and vinblastine instead show a non-linear change in J_max_. Altogether, while transporter expression-activity relationships (EARs) have been experimentally explored in vitro, it cannot yet be concluded that this relationship is always linear.

Our study adds a computational aspect to the study of transporter EARs, with the P-gp kinetic binding model allowing a theoretical exploration of how changes in P-gp expression influence P-gp activity. Our simulations show that whenever the available P-gp expression changes, the relative change in activity does not necessarily scale linearly. For the set of seven drugs that were simulated, under the same system-specific conditions, only 2 showed a distinctly linear P-gp EAR (Fig. 3). For the other compounds this was non-linear: even though P-gp expression changed, P-gp activity remained similar over a large range of P-gp expressions. Only for substantially lower P-gp expressions did the activity start to change.

Importantly, we found that P-gp EARs are strongly impacted by the ratio of the drug P-gp dissociation and drug efflux rate constants (*k*_*off*_*/k*_*e*_*)*, named P-gp efflux efficiency by Tran et al. This ratio indicates how many drug molecules should bind to P-gp, before one is successfully effluxed [36]. Drugs that are inefficiently effluxed (i.e., having a high *k*_*off*_/*k*_*e*_ ratio) are more likely to show linear P-gp EARs, whereas efficiently effluxed drugs show non-linear P-gp EARs. Intuitively, this makes sense: when it is difficult for P-gp to efflux a given compound, it might benefit from an increase in P-gp expression. Overall, we conclude that the common assumption of P-gp activity being directly proportional to its expression in a drug-independent fashion is too simplistic, especially when used to scale a single measure of P-gp activity like the ER between different systems. This is not only supported by the drug-specific properties, but also by the finding that the initial system-specific conditions (P-gp expression and drug concentration) influence the P-gp EAR.

A more dynamic P-gp EAR that can be both linear and non-linear is in line with both in vitro as well as in vivo findings. Our simulations using parameter estimates by Lumen et al. for quinidine, verapamil, and vinblastine showed a linear P-gp EAR at 1 µM drug concentration for verapamil, whereas the other drugs showed non-linear P-gp EARs. This matches the trends observed for the J_max_ in the in vitro results by Tachibana et al. [50] for these drugs (supplementary Figure S6). Also, the different P-gp EARs in Fig. 3 match results reported in vivo at the blood-brain-border (BBB): in line with our quinidine simulations showing a non-linear P-gp EAR, Braun et al. and de Lange et al. found that different BBB P-gp expression levels were not related to brain distribution of quinidine in dogs and rats, respectively [29, 30]. On the other hand, a report on BBB P-gp expression changes correlating well with verapamil efflux in rats [51], is in line with our verapamil’s simulated linear P-gp EAR. Furthermore, Kalvass et al. showed that, for loperamide, complete inhibition of mouse BBB P-gp leads to a ~ 60-fold increase in its K_p,brain_ [52]. However, a 50% inhibition of P-gp leads to a less than 2-fold increase in K_p_,_brain_, indicating a distinctly non-linear P-gp EAR in this scenario. Simulations of loperamide at 1 µM (Fig. 3) and at a lower concentration of 0.1 µM (approximately 50 ng/mL as in Kalvass et al. [53]) show that the rEAR_50%_ is non-linear (supplementary Figure S7). Lastly, Sadiq et al. show that a 10-fold decrease in P-gp expression at the mouse BBB hardly influences the K_p,uu,brain_ of digoxin (0.002 versus 0.004), whereas it does influence the K_puubrain_ of verapamil (0.1 versus 1.6) [54]. This drug-specific P-gp EAR is also shown for these drugs in Fig. 3. Overall, the simulations performed using the parameter estimates by Lumen et al. with 1 µM apical drug concentration and 1000 µM initial P-gp concentration can capture the general drug-specific trends that have been reported in multiple in vitro and in vivo studies.

This study has several limitations. Most significant is that this is a simulation-based theoretical analysis, and all results are specific to the kinetic model and its underlying assumptions. Additionally, simulations do not constitute proof, requiring further experimental validation. These assumptions also warrant further discussion. For example: (1) Is the association rate (*k*_*on*_) indeed drug independent as assumed in the model? [36, 38]; (2) Can the kinetic parameters be uniquely identified? (is what is being fitted not just a certain combination of the model parameters instead?) [55]. These are important questions to consider should the kinetic model be applied to PBPK models. However, the fact that our model simulations capture trends observed in vivo at the BBB, for multiple drugs, supports our conclusions on the P-gp EAR. Another limitation with regards to further implementation of the kinetic binding model, is the assumption that P-gp expression in the apical inner leaflet represents the efflux active concentration, which may deviate from total P-gp expression [36, 37]. As in vitro monolayers have microvilli, only P-gp expressed at the tips are efflux active. How this efflux active P-gp expression (e.g., µM) relates to common measures of P-gp expression (e.g., fmol/µg protein), remains to be determined. The first step in that direction is to report the concentration of P-gp in the plasma membrane of cells as done by Mlejnek et al. [56]. This study reported P-gp membrane concentrations ranging from 155 µM to 1515 µM across five drug-resistant human leukemia K562 cell types. The 1000 µM P-gp expression as estimated by Lumen et al. for MDCKII-MDR1 matches this range well.

The P-gp kinetic binding model has only been applied to fitting in vitro data, and now to investigate the behaviour of P-gp with regards to the EAR. Ultimately, the goal of more advanced models like the kinetic model is to improve our fundamental understanding of transporter mechanics, and as such to improve our translational capabilities. Future studies that incorporate the kinetic binding model within PBPK models for temporal predictions of P-gp mediated disposition will have to show whether the model indeed allows for more robust translation of P-gp activity than more conventional methods.

## Conclusions

Thoroughly understanding the relationship between P-gp expression and activity is crucial for successful *in vitro-in vivo* extrapolation. A common assumption is that P-gp (or transporter) expression is linearly related to its activity. Although some studies have shown a drug independent, linear relationship between expression and activity, many other studies have not been able to identify this. This simulation study shows that the P-gp EAR depends on the drug, drug concentration and initial P-gp expression. A non-linear P-gp EAR is expected when a drug is efficiently effluxed and/or when P-gp is in excess compared to the drug. The a priori assumption that P-gp activity scales linearly with P-gp expression might therefore be correct for some but not for all drugs and scenarios. This is a crucial consideration not only for in vitro informed PBPK predictions of CNS PK, but for predictions of transporter-mediated PK in general.

## Supplementary Information

Below is the link to the electronic supplementary material.

Supplementary File 1 (DOCX 2.48 MB)

## Data Availability

All input data used for model simulations is defined in the main text and derived from previously published research. Raw model output can be obtained upon reasonable request.
