# Supplementary material for: Simulation-based assessment of the P-glycoprotein expression-activity relationship shows a drug and system dependency
Source: J Pharmacokinet Pharmacodyn. 2026 Feb 2;53(2):10. doi: 10.1007/s10928-025-10015-6 (PMC12864343; doi:10.1007/s10928-025-10015-6)
Supplement: Supplementary file 1 — (DOCX 2.48 MB) [file 10928_2025_10015_MOESM1_ESM.docx]

**Supplementary materials to:**

Simulation-based assessment of the P-glycoprotein expression-activity relationship shows a drug and system dependency
Authors: Daan W. van Valkengoed^1^, Vivi Rottschäfer^2,3^, Elizabeth C.M. de Lange^1*^

**Affiliations:**
1: Division of Systems Pharmacology and Pharmacy, Leiden University, The Netherlands
2: Mathematical Institute, Leiden University, The Netherlands
3: Korteweg-de Vries Institute for Mathematics, University of Amsterdam, The Netherlands

*Corresponding author (email: [ecmdelange@lacdr.leidenuniv.nl](mailto:ecmdelange@lacdr.leidenuniv.nl))
Gorlaeus Laboratories, Einsteinweg 55, 2333 CC Leiden, The Netherlands
Tel : +31 71 527 6330


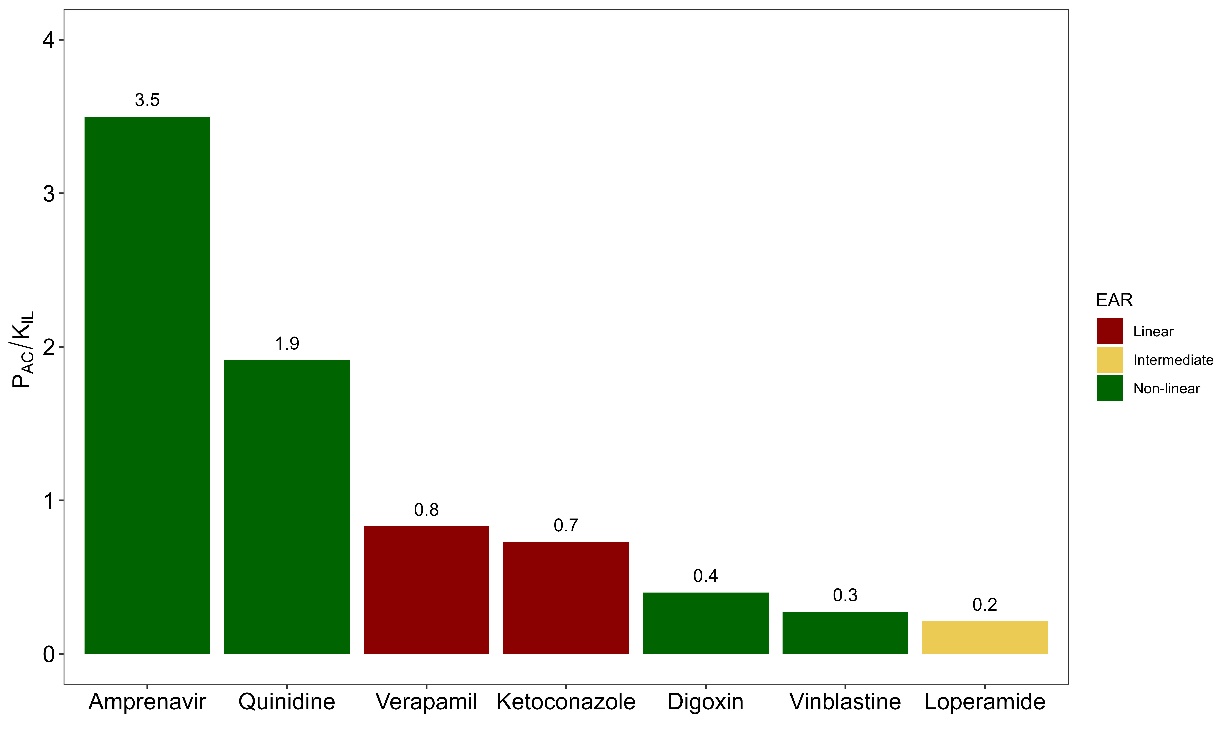
Supplementary figure S1

**Supplementary Figure S1: The relationship between the ratio P_AC_/K_IL_ and the EAR of the P-gp substrates.** Red bars indicate drugs that show a linear EAR (verapamil and ketoconazole), green bars drugs with a non-linear EAR (amprenavir, digoxin, quinidine and vinblastine), and yellow bars a drug with a EAR in between the other two groups (loperamide). EAR was determined through simulations that started with a reference (100%) P-gp expression of 1000 µM and a drug concentration at t = 0 of 1 µM.

#
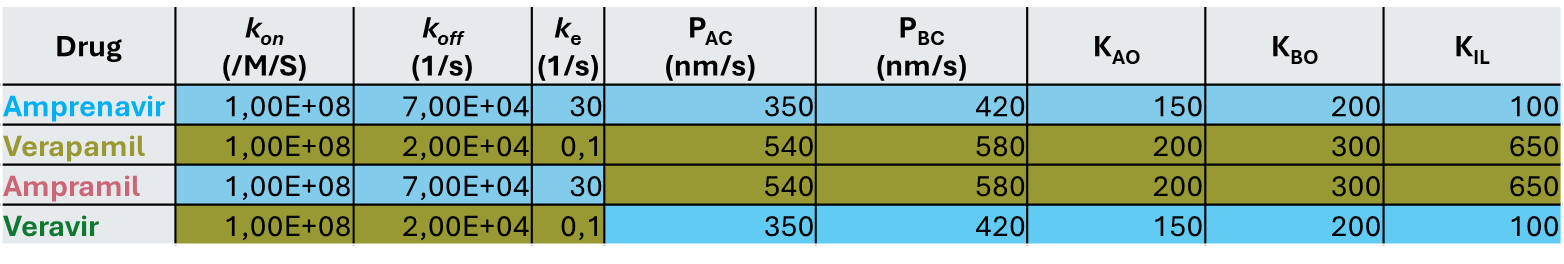
Supplementary Table S1 and Supplementary Figure S2

**Supplementary Table S1: Input parameters for amprenavir, verapamil, and their hybrid drugs ampramil and veravir.** For EAR simulations, see supplementary figure 2.


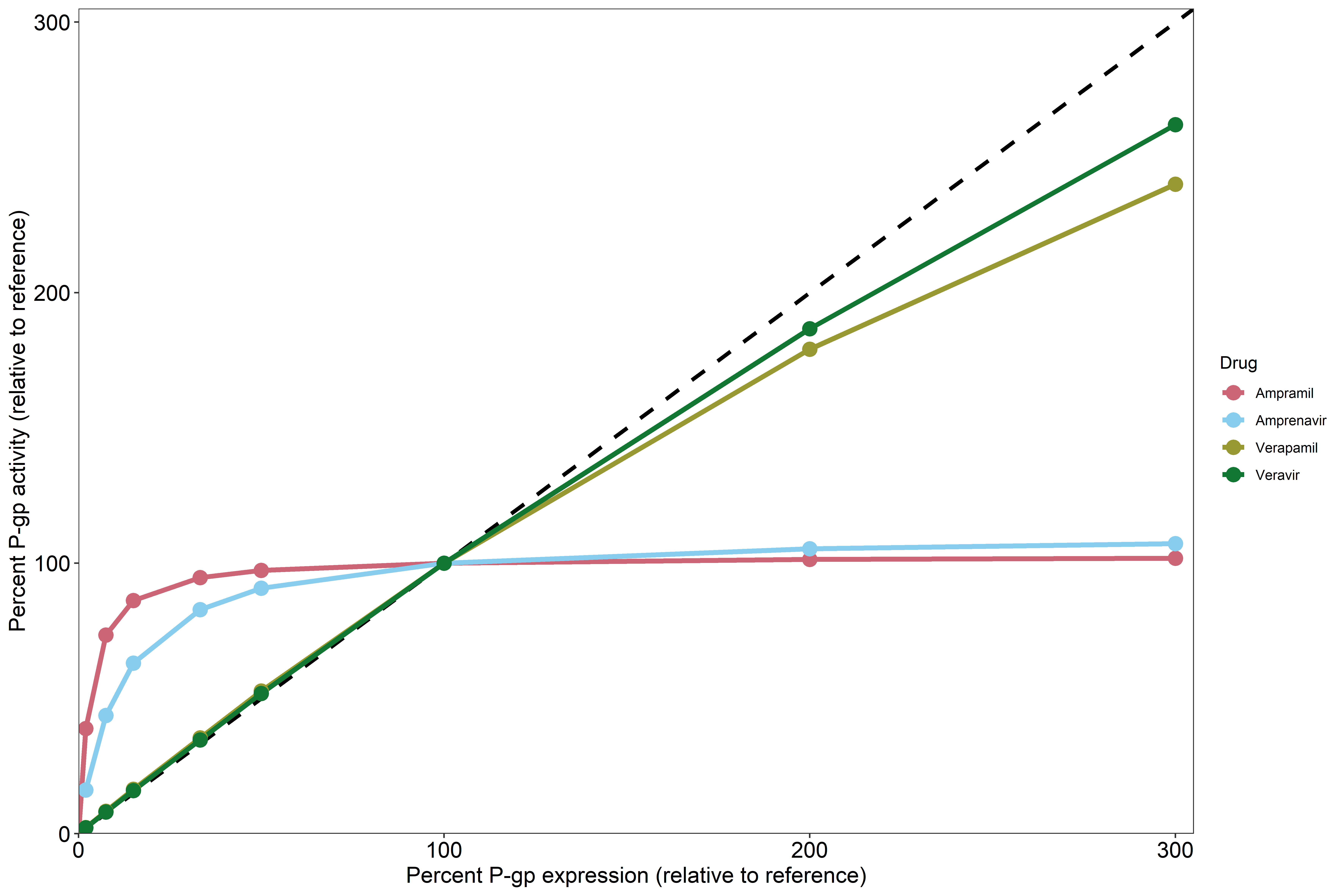


**Supplementary Figure S2: Expression-activity relationship for amprenavir, verapamil and their hybrid drugs ampramil and veravir.** Ampramil and veravir represent compounds with the exact same kinetic parameters as amprenavir and verapamil, but with the passive permeability and partitioning coefficients of the other compound (see supplementary table 1). All the simulations started with a reference P-gp expression of 1000 µM and the drug concentration at t = 0 was 1 µM. The black dashed line indicates the expression and activity of P-gp are proportional.

#



Supplementary Figure S3

**Supplementary Figure S3: Heatmap of the rEAR50% of virtual drugs varying in k_off_ and k_e,_ dosed at 1 µM and with a reference P-gp expression of 1000 µM.** A) All simulated virtual drugs have a passive permeability P_AC_ = 300 nm/s and K_IL_ of 300. B) All simulated virtual drugs have a P_AC_ of 700 nm/s and K_IL_ of 300. In both subplots, red colours indicate a linear response in P-gp activity to the change in P-gp expression (rEAR50% ≈ 50%), while green corresponds to non-linear changes in activity (rEAR50% ≈ 100%). In these simulations, P_AC_ = P_BC._

**B**

**A**

#



Supplementary Figure S4

**A**

**B**

**Supplementary Figure S4: Heatmap of the rEAR50% of virtual drugs varying in k_off_ and k_e,_ dosed at 1 µM and with a reference P-gp expression of 1000 µM.** A) Simulations done using passive permeability P_AC_ = 300 nm/s and K_IL_ of 300. B) Simulations done using P_AC_ of 300 nm/s and K_IL_ of 700. In both subplots, red colours indicate a linear response in P-gp activity to the change in P-gp expression (rEAR50% ≈ 50%), while green corresponds to non-linear changes in activity (rEAR50% ≈ 100%). In these simulations, P_AC_ = P_BC._

#
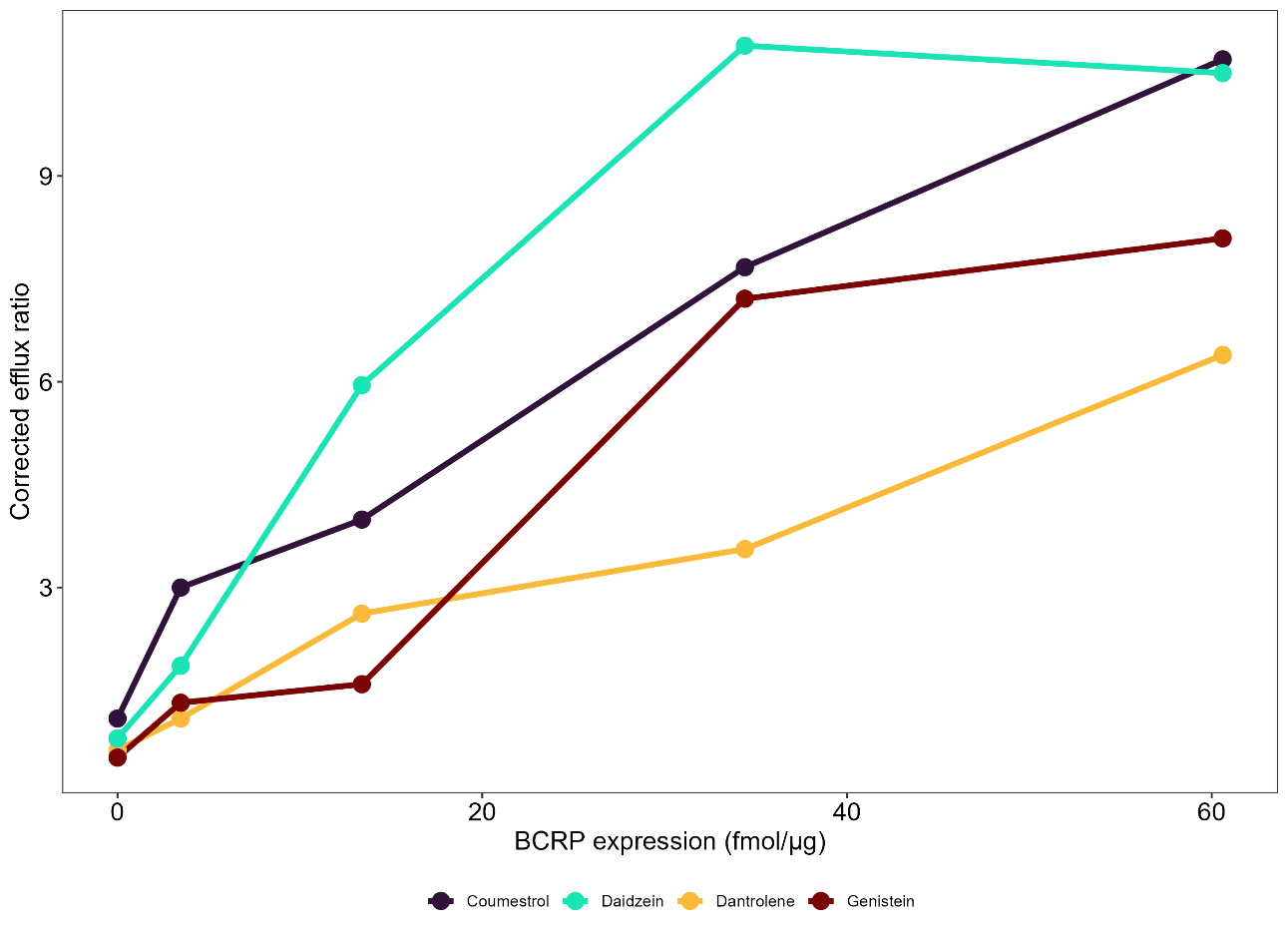
Supplementary Figure S5

**Supplementary Figure S5: BCRP EAR for Coumesterol, Daidzein, Dantrolene and Genistein extracted from Liu et al. [1].**

#
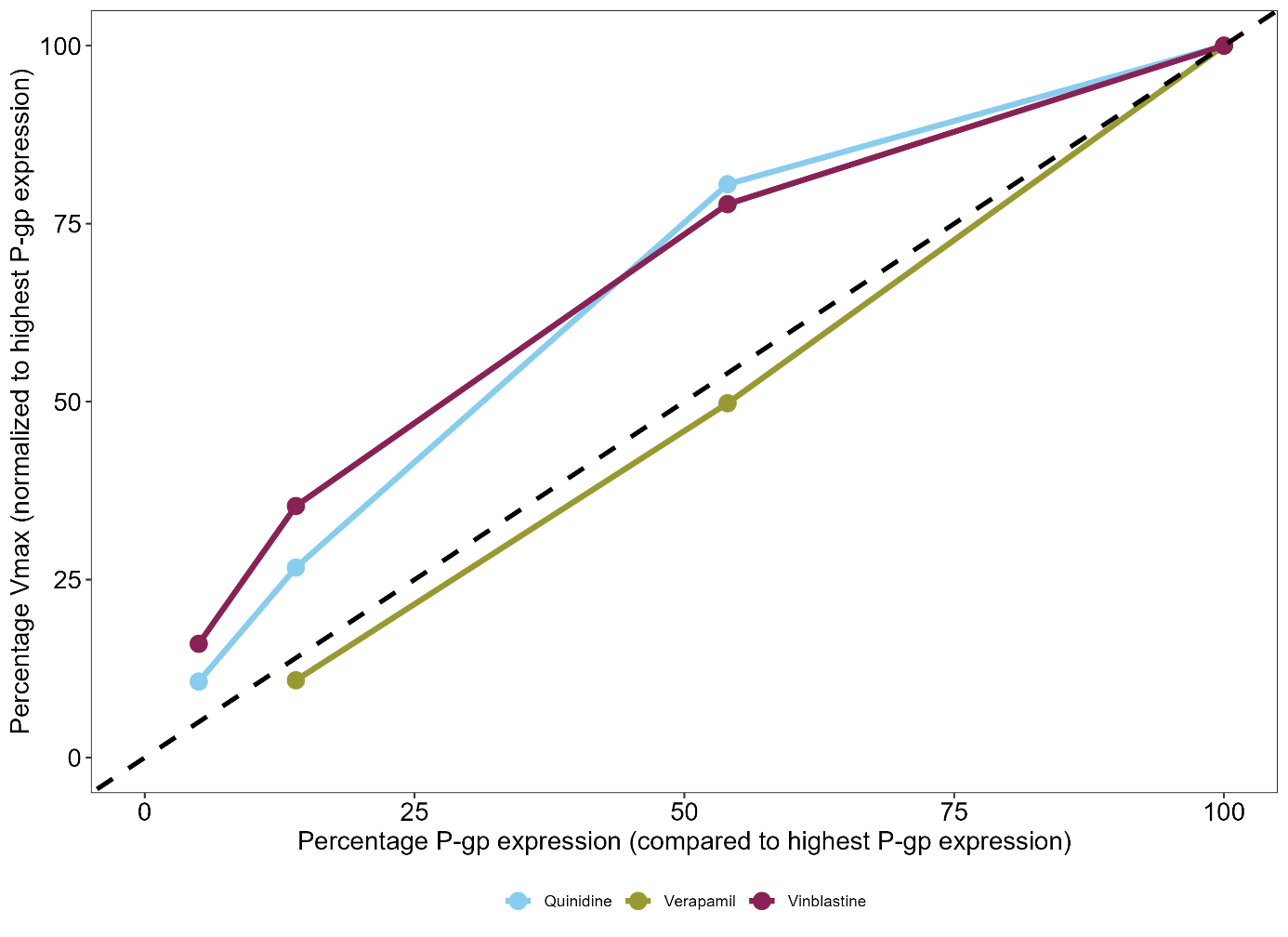
Supplementary Figure S6

**Supplementary Figure S6: Normalized data of P-gp EAR for quinidine, verapamil and vinblastine extracted from Tachibana et al. [2]. Data shown here corresponds to the data measured in the Caco-2 cell lines only.** Only Caco-2 derived data was plotted to ensure the data come from the same system which might influence the relationship. This is visible in the drug-specific parameters in these systems: the passive permeability was higher in MDCKII cells (quinidine = 34.0 * 10^-6^ cm/s) than in Caco-2 (average for quinidine = 26.6 * 10^-6^ cm/s, n = 4), as well as the estimated K_m_ (0.339 µM versus 0.229 µM (average, n = 4) for quinidine). The expression of P-gp is originally given in µg P-gp per cm^2^. The data is expressed relative to the highest P-gp expression in the Caco-2 cells of 191 µg/cm^2^. The percentage J_max_ was then calculated as: $\frac{J_{max} at expression x \mu g/{cm}^{2}}{J_{max} at 191 \mu g/{cm}^{2}}*100\%$for each drug.


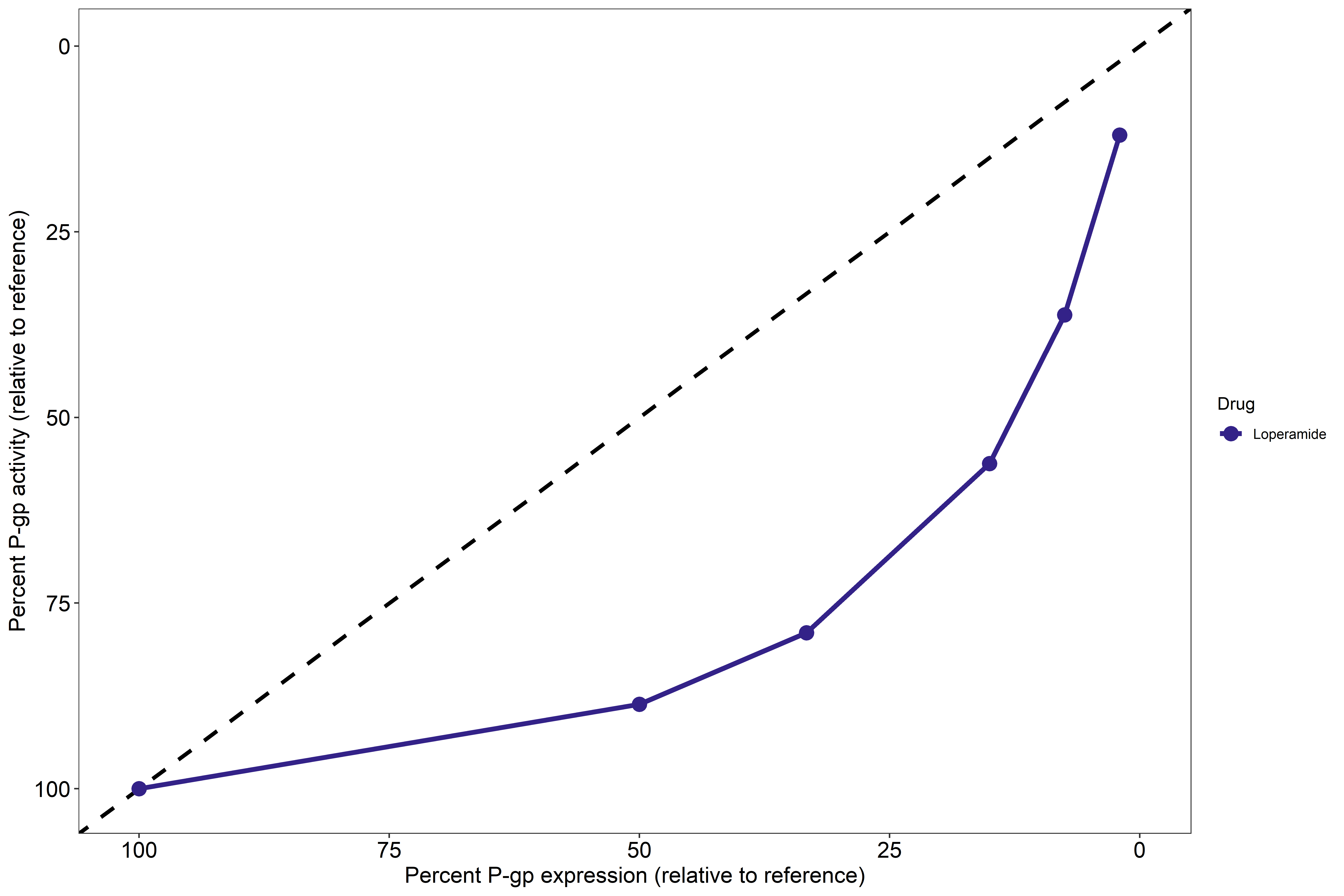
Supplementary Figure S7:

**Supplementary Figure S7: Loperamide EAR determined at an initial drug concentration of 0.105 µM and reference P-gp expression of 1000 µM.** The dose of 0.105 µM was chosen as it corresponds to the plasma concentration of approximately 50 ng/mL as described Kalvass et al. [3]. The plot is shown with highest expression and activity starting at the origin to allow easier comparison to the figure published in Kalvass et al. [3].

# References

1. Liu, H., et al., *Correlation between Membrane Protein Expression Levels and Transcellular Transport Activity for Breast Cancer Resistance Protein.* Drug Metab Dispos, 2017. **45**(5): p. 449-456.

2. Tachibana, T., et al., *Model analysis of the concentration-dependent permeability of P-gp substrates.* Pharm Res, 2010. **27**(3): p. 442-6.

3. Kalvass, J.C., C.L. Graff, and G.M. Pollack, *Use of loperamide as a phenotypic probe of mdr1a status in CF-1 mice.* Pharm Res, 2004. **21**(10): p. 1867-70.
